# Effect of Dietary Polyphenols on Osteoarthritis—Molecular Mechanisms

**DOI:** 10.3390/life12030436

**Published:** 2022-03-16

**Authors:** Mateja Sirše

**Affiliations:** Department of Orthopaedics, University Medical Centre Maribor, Ljubljanska Street 5, 2000 Maribor, Slovenia; mateja.sirse@gmail.com

**Keywords:** osteoarthritis, plant polyphenols, molecular mechanisms, inflammation, oxidative stress, signaling pathway, cartilage, chondrocytes, synovium

## Abstract

Osteoarthritis is a common crippling and degenerative disease resulting in irreversible functional changes due to damage of the cartilage and other tissues of the joint. With limited safe and effective pharmaceutical treatments, the demand and use for alternative therapeutic approaches with symptomatic relief for OA patients have increased. Clinical, pre-clinical, and in vitro studies have demonstrated that polyphenols can exert pain-relieving symptoms coupled with increased functional capacity in OA models. This review will highlight studies carried out in the last five years to define the efficacies and underlying mechanisms in polyphenols such as quercetin, resveratrol, curcumin, epigallocatechin-3-gallate, rosmarinic acid, genistein, ginger, berries, silver fir, pine bark, and *Boswellia*. Most of these studies indicate that polyphenols exhibit their beneficial roles through regulating changes at the biochemical and molecular levels, inducing or inhibiting various signaling pathways related to inflammation and oxidative stress. Polyphenols have also been implicated in modulating microRNA at the posttranscriptional level to counteract OA pathogenesis.

## 1. Introduction

Osteoarthritis (OA) is a common and chronic inflammatory degenerative disease affecting millions worldwide. This pathology is characterized by slow, progressive damage and loss of the articular cartilage and periarticular muscle, coupled with the formation of osteophytes, synovial inflammation and degeneration of ligaments, subchondral bone, menisci and infrapatellar fat pad, which further leads to the formation of osteophytes, subchondral sclerosis, bone cysts, and narrowing of the joint space [1,2,3]. The most prevalent risk factors of primary OA are aging, genetic predisposition, sociodemographic characteristics, and diet-related factors [1]. The latest report reveals that 7% of the global population, or more than 500 million people, have been diagnosed with OA [1,2,3].

There have been many efforts made to alleviate the symptoms and slow the progression of this disease. However, OA has no cure. The widely used conventional treatments have shown the benefits to be limited and restricted primarily to the use of analgesics, nonsteroidal anti-inflammatory drugs (NSAIDs), corticosteroid injections, and joint replacement as an effective alternative [4]. These treatments only provide temporary systemic relief and not cures, with documented adverse side effects and toxicity [5,6]. Nevertheless, there has been some progress in understanding the molecular pathophysiology of OA using high-throughput genomics technologies. A variety of potential therapeutic targets of OA have been identified; these include identifying signaling pathways and the involvement of critical genes and microRNAs (miRNAs) in various musculoskeletal disorders [7].

Osteoarthritis is a whole joint disease where all or almost all joint tissues are involved and/or affected by this degenerative process [1,3]. OA is characterized by a metabolic imbalance between anabolic and catabolic factors produced by chondrocytes, which leads to cartilage degradation and destruction. A large body of evidence supports the role of inflammation in OA and the involvement of the synovium, infrapatellar fat pad, and other joint structures, which secrete inflammatory cytokines [3]. Changes in subchondral bone and muscles further contribute to the increase in joint loading [1,2]. Chondrocytes located in articular cartilage serve a key role in ensuring matrix integrity and become activated and undergo phenotypic instability in response to local and systemic injuries, thus contributing to the irreversible damage of articular cartilage [8]. In damaged joints, the degraded products from the cartilage and the secreted inflammatory factors from chondrocytes stimulate synovial inflammation in OA [9]. Proinflammatory cytokines such as IL-1β, tumor necrosis factor (TNF)-α, and IL-6 are critical mediators of articular cartilage degeneration and synovium inflammation [9,10,11]. In other studies, the nuclear factor-kappaB (NF-κB) and the mitogen-activated protein kinase (MAPK) have been suggested as alternative treatments for OA [9,12].

There is also a close association between OA progression and oxidative stress. Oxidative stress and an imbalance in the pro-oxidant/antioxidant status can result in elevated reactive oxygen species (ROS) production in OA cartilage and chondrocytes [13]. Consequently, therapy targets the degradation of the cartilage resulting from the lipid peroxidation and is evident with the presence of peroxidation products in OA tissues [14].

The involvement of the anti-inflammatory cytokines and oxidative stress suggests that the modulation of these cellular responses by adding appropriate antioxidants and anti-inflammatory agents could represent a viable and safe strategy for treating OA patients [2]. Numerous studies have revealed that polyphenols, including anthocyanins, phenolic acids, and flavonoids, which are abundantly found in herbs, fruits, and vegetables, have potential therapeutic potential in modulating the progression of OA. This review article will summarize the scientific evidence and possible mechanisms of action of polyphenols for OA treatment.

The current review mostly highlights the published articles implicating the pharmaceutical and therapeutic effects of polyphenols on OA, with particular emphasis on the underlying molecular mechanism associated with oxidative stress, pain, and inflammatory disorders. The data (Google Scholar, PubMed, and Scopus) search was carried out by examining related keywords such as, “osteoarthritis”, “plant polyphenols,” “quercetin,” “curcumin/curcuminoid”, “resveratrol”, “epigallocatechin-3-gallate and green tea polyphenols”, ”rosmarinic acid”, “genistein”, “strawberries”, “blueberries”, “silver fir”, “pine bark”, “boswellia”, “ginger”, “analgesic”, “antinociceptive”, “inflammation”, “anti-inflammatory”, “molecular mechanisms”, “inflammation”, “oxidative stress”, “signaling pathway”, “cartilage”, “chondrocytes”, “synovium”, “infrapatellar fat pat”, “ligaments”, and “meniscus”. 

## 2. Molecular Mechanism of OA

Insights into recent molecular evidence revealed that OA is not only limited to a simple degenerative “wear and tear” disease of the cartilage, as previously thought. OA is a multifactorial and degenerative joint disease driven by multiple-signal transduction pathways, working together in the entire joint (involving tissues in or around the joint) that eventually results in an imbalance between the catabolic and anabolic processes. This balance is vital for cartilage tissue integrity and repair, and, when compromised, it can cause tissue degradation leading to pain, stiffness, and physical disability [15]. Basically, OA affects the articular cartilage (AC), subchondral bone, the synovium (synovial membrane and synovial fluid), Hoffa’s infrapatellar fat pad (IPFP), periarticular muscles, ligaments, and tendons [15].

The onset of OA is mainly due to aging but can be caused by circumstances that precipitate cartilage aging, such as metabolic syndrome and obesity [16]; the entire joint undergoes marked changes, and the ability to adapt to stress is significantly reduced. AC comprises of chondrocytes and a dense extracellular matrix (ECM). More than 70% of the ECM consists of water, and the rest consists of organic components such as collagen type II (COL-II), aggrecans, proteoglycans, glycosaminoglycans, and glycoproteins [15]. In the progression of OA, an increase in water content in the articular cartilage coupled with a loss of glycosaminoglycans and proteoglycan degradation is observed [15]. This is due to the disparity in the cytokine balance between concentrations of both proinflammatory and anti-inflammatory cytokines. Excessive proinflammatory cytokines lead to inflammation, cartilage degradation, and synovial proliferation, thus leading to OA [17]. 

Under biomechanical stress and injury, chondrocytes and synoviocytes produce inflammatory cytokines and chemokines in the synovial fluid [18]. One of the essential proinflammatory cytokines involved in the pathogenesis of OA is IL-1β. A cascade of signaling pathways associated with OA progression is activated after binding to the receptor (IL-1RI). Interleukin (IL)-6 is another significant proinflammatory cytokine associated with the pathogenesis of OA. However, its exact function is not clearly defined, as there have been contradictory results on the effects of IL-6 on the progression of OA in animal models [19,20]. In human studies, the role of IL-6 seems to be more definitive; OA patients at their end-stage have a significantly higher IL-6 concentration in synovial fluid compared with healthy individuals [21]. 

Another major pathway activated by IL-1β is the NF-κB signaling pathway; other than inducing catabolic enzymes, the activation of NF-κB induces the expression of major proinflammatory mediators of OA such as inducible nitric oxide synthase (iNOS), cyclooxygenase 2 (COX-2), and prostaglandin E2 (PGE2) [9]. NF-κB activation also leads to the inhibition of type II collagen expression and increases the production of matrix metalloproteinases (MMPs) and aggrecanases. Additionally, the IL-1β-activated NF-κB pathway stimulates the synthesis of proinflammatory cytokines and secretion, such as IL-6 and TNF-α [18]. 

The degradation in OA pathogenesis is due to the aggrecanases of ADAMTS, particularly ADAMTS-4 and ADAMTS-5 and the MMPs such as MMP-13, MMP-1, MMP-2, MMP-3, MMP-7, MMP-8, and MMP-9 [22]. This process causes an accumulation of advanced glycation end-products (AGEs) [22]. MMP-13 has been implicated as the main expressed proteinase in OA due to its ability to degrade COL-II, the cartilage’s main structural protein [23]. Furthermore, MMPs coupled with endogenous tissue inhibitors of metalloproteinases (TIMPs) play a vital role in the homeostatic balance of the matrix anabolism and catabolism [24]. AGE build up causes alteration in the biomechanical properties of cartilage, causing pathologic stiffening of the cartilage and ECM [25]. AGEs also bind to specific cell surface receptors (such as RAGE) and alter gene expressions and multiple-signal transduction pathways involving the activation of numerous inflammatory pathways and production of reactive oxygen species (ROS) and oxidative stress [26]. 

The IL-1β-mediated NF-κB activation has also been reported to increase the expression of chemokines such as IL-8, monocyte chemoattractant protein-1 (MCP-1 or CCL2), CCL5, and macrophage inflammatory protein-1a (MIP-1a), causing inflammation of the synovial, which in turn further stimulates the production of IL-1β [9]. 

TNF-α is another potent proinflammatory cytokine and, along with IL-6 and IL-1β, acts as a catabolic factor for the cartilage [17]. TNF-α, once bound to its receptor (TNRF-1 and TNRF-2), activates the mitogen-activated protein kinases (MAPK) and the NF-κB, inducing a series of catabolic processes involving cartilage degradation, cell death, or apoptosis and inhibits chondrogenesis [17]. MAPK entails three different families, namely the extracellular signal-regulated kinases (ERKs), c-Jun N-terminal kinases (JNKs), and p38 MAPKs, all of which are involved in OA [27]. As in IL-1β, TNF-α can also play a role in ECM degradation by inducing the production of MMP-1, MMP-3, MMP-13, and ADAMTS-4 [17,28].

With the rapid advancement and utilization of high-throughput genomics technologies, numerous microRNAs (miRNAs) that modulate gene expression at the posttranscriptional level are implicated in the initiation and progression of OA [29]. Marked changes in the miRNome are observed in OA’s pathogenesis, reviewed comprehensively by Endisha [30]. More recently, additional miRNAs involved in knee OA have been identified; these include miR-101, miR-181a, miR-29, miR-9, miR-221, miR-411, miR-455, and miR-132 [7,31,32,33]. MiRNAs also regulate Wnt/βcatenin pathways [34], Hypoxia-inducible factor-*1* α (HIF-1) [7], Hypoxia-inducible factor-*2* α (HIF-2 α), and PTEN/PI3K/AKT signaling pathways [30,31], all of which are implicated in the pathogenesis of OA. 

## 3. Efficacy and Mechanism of Action of Polyphenols

More than 8000 different polyphenols have been identified in various plant species; they are also abundantly present in beverages, food, spices, and herbs. Polyphenols such as phenolic acids, flavonoids, anthocyanin, stilbenes, and lignans have long generated interest for their potential health benefits. There has been a substantial increase in research over the last two decades into the health benefits of polyphenols. They exhibit antioxidant, anti-inflammatory, antimicrobial, anticarcinogenic, antiadipogenic, anti-hypertensive, antidiabetic, and neuroprotective effects [35,36,37]. Their ability to regulate and module immune cells to reduce oxidative stress and inflammation suggests that polyphenols could play a crucial role in beneficially impacting chronic diseases [35,38]. Several studies carried out in the past two decades have found the beneficial role of polyphenols in OA management [39,40,41]. The lower prevalence of OA has been associated with frequent consumption of food rich in polyphenols [41]. Polyphenols can modulate gene expression, activate signaling pathways, play an important role in rescuing altered homeostatic systems, and modulate epigenetic changes [22,39,40,41,42,43,44].

This review will highlight the advancement made using genomics technology over the last five years on commonly consumed polyphenols in modulating the pathogenesis of OA. It will focus on recent investigations on polyphenols such as quercetin, resveratrol, epigallocatechin gallate (EGCG), curcumin, genistein, and herbs or whole plant extracts such as ginger, berries (strawberry and blueberry), silver fir, pine bark, and *Boswellia*.

### 3.1. Quercetin

Quercetin, a naturally occurring flavonol in many vegetables, herbs, and fruits, has been comprehensively investigated for its many varied health benefits over the last few decades. Due to its many pharmacological properties, such as its antioxidant, anti-inflammatory, antiviral, antineoplastic, cardioprotective, and antibacterial activities, quercetin has become a popular nutraceutical used in the food and pharmaceutical industries [45]. 

The therapeutic effects of quercetin on OA’s progression through modulating and inhibiting several signaling pathways both in in vitro and in vivo animal models have been validated (Table 1). Li et al. showed quercetin’s ability to suppress the IRAK1/NLRP3 signaling pathway and consequently decrease the levels of proinflammatory cytokines (IL-1β, IL-18, and TNF-α) in a rat OA model. This was validated in vitro where quercetin suppressed the expression of IRAK1, NLRP3, iNOS, COX-2, and Caspase-3 in IL-1β-induced rat chondrocytes, thus, exhibiting quercetin’s ability in attenuating inflammation, apoptosis, cartilage degradation, and reducing oxidative stress in rat chondrocytes [46]. Consistent with these findings, Hu et al. also demonstrated that quercetin exerts chondroprotective effects reducing cartilage degradation and apoptosis of chondrocytes in the rat OA model [47]. In this study, the expression of the Caspase-3 pathway was also inhibited; the expression of matrix-degrading proteases was downregulated, and the cartilage matrix degradation was reversed in IL-1β-induced rat chondrocytes. In addition, the expression of transforming growth factor β (TGF-β1 and TGF-β2) was upregulated in the synovial fluid. Quercetin also induced M2 polarization of macrophages, which created a pro-chondrogenic microenvironment for chondrocytes and promoted the synthesis of glycosaminoglycan to enhance cartilage repair [47]. In yet another recent study, quercetin at a concentration of 100 µmol/l was shown to inhibit the p38 MAPK signaling pathway and ADAMTS, decreasing relevant inflammatory factors in the pathogenesis of OA and promoting the expression of COL-Ⅱ to initiate cartilage repair [48]. Quercetin also exhibited its anti-OA properties by inhibiting MMP, indicated by a significant reduction in the serum MMP-3 and MMP-13 concentrations in a monosodium iodoacetate (MIA)-induced knee mouse OA model [49].

### 3.2. Resveratrol

Resveratrol, a stilbene, is another polyphenol found abundantly in grape skins, red wine, peanuts, and Japanese knotweed [66]. This nutraceutical has many health-benefiting properties, including its anti-inflammatory, antioxidant, and anticancer properties. By modulating major oxidative stress signaling pathways, resveratrol has become one of the most studied nutraceuticals in managing degenerative diseases such as OA [51,52,54,55,56,57,67] (Table 1). 

Two recent clinical studies confirmed the involvement of resveratrol in managing knee OA pain and its associated symptoms [51,67]. Supplementation of 500 mg/day resveratrol led to increased aggrecan serum levels and significantly improved pain, although no significant decrease in serum levels of IL-6, IL-1β, and TNF-*α* was observed [56]. Furthermore, in an in vitro study conducted on monocytic cell line (THP-1) resveratrol and its natural precursor polydatin inhibited ROS and NO production and IL-1 and IL-1β mRNA expression induced by monosodium urate and calcium pyrophosphate crystals. This study demonstrated that resveratrol and its precursor can decrease inflammation induced by calcium pyrophosphate crystals, which can also be present in OA joints and seem to be involved in synovial inflammation [54].

In vivo and in vitro studies provided more detailed mechanistic information on the resveratrol’s actions in modulating pain, reducing inflammation, and exerting a protective effect in OA patients [51,52,55,57]. Its protective effects were demonstrated in an in vivo model where resveratrol ameliorated inflammatory damage in a rat OA model [55] through its impact on the NF-κB and HO-1/Nrf-2 signaling pathway [52]. Resveratrol downregulated the expression of TNF-α, IL-1β, IL-6, and IL-18 and the caspase-3/9 activity. In addition, it also suppressed the activity of iNOS, and reduced the expression of nuclear factor (NF)-κB and phosphorylated-(p)-AMP-activated protein kinase and sirtuin 1 (SIRT 1) protein [55]. The expression of HO-1 and Nrf-2 was upregulated in resveratrol-treated OA rats [55]. The mechanism of resveratrol in reducing soft tissue damage in a rat OA model was deciphered using network pharmacological analysis; results revealed that resveratrol can inhibit the TLR4-mediated NF-κB signaling pathway and can reverse the damage of soft tissue in OA. This is evident through a significant reduction in the IL-1β, IL-6, IL-17, TNF-α, and MCP-1 serum levels, the decrease in the protein levels of TLR-4 and NF-κB p65, and the reduction in the caspase-9 and Bax protein levels [56].

In vitro studies further validated the role of resveratrol in managing OA pathogenesis. Resveratrol reduced the oxidative stress-induced response in human osteoarthritic chondrocytes [35] and reduced inflammation in IL-1β-induced SW1353 chondrocytes cells through inhibiting the TLR4/NF-κB signaling pathway [52]. In hydrogen peroxide-induced OA-like cell models, its mode of action in decreasing inflammation was through the inhibition of TNF-α and the IL-1β and MMP-1 [53]. 

### 3.3. Curcumin/Curcuminoid

Curcuminoids are bioactive compounds found in turmeric (*Curcuma longa*). Curcumin is the most researched type of curcuminoid due to its potent antioxidant, anti-inflammatory, antimicrobial, antirheumatic, neuroprotective, anticancer, hepatoprotective, and cardioprotective properties [68]. Several studies have reported the pain-alleviating properties and the chondroprotective effects of curcumin and curcuminoids in clinical, in vivo, and in vitro studies [58,59,60,61,62,63,64] (Table 1). 

Clinical studies revealed that a curcuminoid-rich extract reduced OA-associated pain but did not affect cartilage composition or knee effusion–synovitis [59]. Another study explored the potency of curcuminoids supplemented with diclofenac in pain reduction. The pain was alleviated with greater functional capacity, and adverse effects were significantly reduced [60]. In an attempt to increase the bioavailability and potency of curcumin, surface-controlled water-dispersible curcumin (Theracurmin) was developed and tested on OA patients [58]. A daily intake of 180 mg of curcumin/ Theracurcumin effectively reduced pain in 76.9% of the OA patients [58]. 

Numerous in vivo models have validated the analgesic effects of curcuminoids and, in addition, provided mechanistic insights into their chondroprotective effects in an OA-induced rat model [61,62,63]. For example, curcumin supplementation in rats fed with a high-fat diet for 28 weeks inhibited apoptosis via increasing the expression of E2F1/PITX1; it also upregulated autophagy through the Akt/mTOR pathway by reducing the microRNA-34a [61]. In another recent study, a highly bioavailable formulation of curcumin (64.7 times more bioavailable) was tested for its protective effects against monosodium iodoacetate (MIA)-induced knee OA in rats [62]. X-ray and histopathological images show that this novel formulation reduced inflammation, decreased joint swelling, and reinstated joint architecture induced by MIA. The results were further validated through biochemical and molecular studies, which show that curcumin significantly reduced levels of TNF-α, IL-1β, IL-6, COMP, and CRP in the synovial tissue of rats. This was accompanied by an increase in the antioxidant enzymes SOD, CAT, and GPX, accompanied by a reduction in the expressions of MMP-3, 5-LOX, COX-2, and NFκB. In addition, the serum MDA levels were decreased [62]. 

Curcumin has been reported to play a vital role in attenuating endoplasmic reticulum (ER) stress-associated apoptosis, as demonstrated in an anterior cruciate ligament transection (ACLT) rat OA model [63]. Curcumin at 50 mg/kg and 150 mg/kg reduced the articular cartilage degeneration inhibited chondrocyte apoptosis and ER stress in the ACLT rat OA model. In vitro, curcumin inhibited the PERK-eIF2α-CHOP axis of the ER stress response and enhanced the expression SIRT 1 in tert-butyl hydroperoxide-induced chondrocytes [63]. Another in vitro study also confirmed the anti-inflammatory effects of curcumin, where IL-1β-induced inflammation was reduced and the mRNA expression of COL-II and SOX9 in chondrocytes were downregulated. Further immunofluorescence and immunohistochemistry studies revealed that curcumin drastically reduced the activation of NF-κB/HIF-2α in IL-1β-induced chondrocytes [64].

### 3.4. Epigallocatechin-3-Gallate and Green Tea Polyphenols

Green tea is rich in polyphenols such as catechin, epicatechin (EC), epicatechin-3-gallate (ECG), epigallocatechin (EGC), and epigallocatechin-3-gallate (EGCG). Of these, EGCG is the most abundant polyphenol in green tea. EGCG coupled with the other green tea polyphenols has exhibited a wide range of biological activities. They possess antioxidant, anticancer, anti-inflammatory, anti-collagenase, and antifibrotic properties [69]. Due to their potent anti-inflammatory and antioxidant properties, green tea polyphenols have gained interest in OA research. Green tea polyphenols were reported to have anti-arthritic and chondroprotective effects through inhibiting inflammation-associated genes, upregulating anabolic mediators, and modulating the expression of miRNAs associated with OA [70,71,72,73,74,75,76] (Table 2).

Over the last five years, there has been limited evidence in testing the efficacy of EGCG and other green tea polyphenols in mitigating cartilage degradation and alleviating pain in joints during OA progression. In an acute gout mouse model, oral administration of EGCG to mice effectively alleviated gout inflammatory symptoms [77], which was previously already seen in vitro [76]. Another study carried out by Hashempur et al. reported that pain was significantly reduced in the intervention group receiving green tea polyphenols plus diclofenac compared to the control receiving only diclofenac. However, no significant differences were observed in the knee stiffness in the treated group [73]. 

EGCG and green tea polyphenols have been shown to decrease inflammation, reduce cartilage degradation, and improve functionality in various animal OA models [70,71,74,75]. In a study using an ACLT mice model, intra-articular injection of EGCG was found to significantly reduce posttraumatic OA via improving functional performances and decreasing cartilage degradation [70]. EGCG reduced inflammation in the cartilage and synovial tissues by inhibiting the production of COX-2 and MMP-13. In addition, EGCG downregulated mTOR expression, upregulating the expression of Beclin1, LC3, and p62, thereby, possibly activating autophagy [70]. Similarly, in another study, an intra-articular EGCG injection aging-related OA model in guinea pigs also ameliorated cartilage degradation with reduced proteoglycan loss and articular surface erosion. EGCG exhibited its anti-OA properties by reducing apoptosis with reduced-immuno-stained COL-II, MMP-13, and p16 ^Ink4a^ [71]. These results were verified in vitro, where EGCG was found to module gene expression by upregulating aggrecan and COL-II expression and downregulating expressions of cyclooxygenase 2, IL-1, MMP-13, alkaline phosphatase, and p16 ^Ink4a^ [71]. In a rabbit OA model, green tea extract via injection significantly decreased knee joint damage coupled with a decrease in NO proinflammatory levels [74]. In a recent investigation, an injectable hydrogel (containing hyaluronic acid) coupled with 50 μM EGCG protected chondrocytes reduced OA progression and ameliorated cartilage loss in a surgically-induced mouse OA model [75]. Further in vitro studies revealed that the administration of this injectable hydrogel reduced the expression of IL-1β, COX-2, TNF-α, MMP1, MMP13, and ADMTS5, and upregulated expressions of COL-II, SOX9, and ACAN, thereby displaying its anti-inflammatory and chondroprotective capacities [75].

The ability of EGCG to modulate miRNA expressions was demonstrated by Rasheed et al. in an IL-1β-induced human OA chondrocyte. EGCG inhibited the inflammatory response through upregulating expression of miR-140-3p, which in turn inhibited ADAMTS5 expression [72].

### 3.5. Rosmarinic Acid

Rosmarinic acid is a polyphenol found abundantly in some plants of the Lamiaceae family, such as rosemary, basil, mint, oregano, lemon balm, and sage. Rosmarinic acid is a widely researched polyphenol due to its exceptionally varied biological activities, ranging from antiviral, antibacterial, antioxidant, anti-inflammatory, antidiabetic, anticancer, and cardioprotective hepatoprotective, nephroprotective, antidepressant, and antiallergic activities [85]. In addition, rosmarinic acid has been implicated for its anti-OA properties, as indicated in Table 2 [78].

In vitro studies established that rosmarinic acid can suppress oxidative stress and reduce the production of NO and PGE2 in IL-1β-induced rat chondrocytes via downregulating the MMP-1, MMP-3, and MMP-13 expression. Furthermore, p38 and JNK phosphorylation and p65 translocation in rat articular chondrocytes were inhibited [79]. In rabbit articular chondrocytes, rosmarinic acid treatment increased differentiation of chondrocytes coupled with increased production of COL-II, sulfated-proteoglycan, COX-2, and PGE2. In addition, rosmarinic activated the ERK-1/2 and p38 kinase signaling pathways [80]. In another study on IL-1β-induced rat chondrocytes, rosmarinic acid inhibited the production of IL-6, downregulated gene and protein expression of ADAMTS-4 and ADAMTS-5, and abolished the IL-1β-induced inhibition of ACAN and COL2 gene expression [81].

### 3.6. Genistein

Genistein, an isoflavone belonging to the sub-class of flavonoids, is one of the most studied isoflavones. This is due to its potent therapeutic properties in alleviating diseases such as diabetes, lipid metabolism, depression, neurodegeneration, bone health, and cardiovascular disease and is associated with its antioxidant, anti-inflammatory, anticancer, and cytotoxic activities [86].

Numerous studies have demonstrated that two estrogen receptors, alpha, and beta, are located in normal and osteoarthritic cartilages, which in turn signifies that cartilage can respond to estrogens [87]. Genistein is a phytoestrogen that shares structural similarities with selective estrogen receptor modulators, and this has prompted studies to explore the effects of genistein in alleviating OA symptoms and on cartilage metabolism [82,83,84] (Table 2).

To delineate the effects of genistein on the pathogenesis of OA, both in vivo and in vitro studies were carried out. The in vivo results revealed that genistein attenuated the progression of OA, decreased chondrocyte apoptosis, slowed cartilage degeneration [82,83,84], and reversed the condyle cartilage damage in an OA-induced rat model [80]. In an in vitro study, genistein reduced inflammation and oxidative stress in IL-1β-stimulated human OA chondrocytes [82]. Genistein exhibited its anti-OA properties through numerous pathways. Liu et al. showed that genistein displayed its beneficial effects through inhibiting the production of NO and downregulating COX-2, MMP-1, MMP-2, MMP-3, and MMP-13. In addition, genistein stimulated the Ho-1 expression and activated the Nrf-2 pathway, reducing IL-1β, IL-6, and TNF-α levels in the serum [82]. In another study, genistein increased COL-II and aggrecan expression levels and downregulated caspase 3 expressions, which resulted in reduced cartilage degradation. This was accompanied by a decrease in the levels of TNF-α IL-1β in the synovial fluid [83]. Genistein treatment has also been shown to reverse the damage of the condylar cartilage via the downregulation of p65 expression and inflammatory cytokines (IL-1β and TNF-α) [84]. 

### 3.7. Ginger

Ginger (*Zingiber officinale* Rosc.) is rich in gingerol. This functional polyphenol is endowed with health-benefiting properties that exhibit a range of biological activities in managing diseases related to inflammation and oxidative stress [88], inclusive of OA (Table 3).

A meta-analysis was conducted on seven clinical studies, and most of the studies indicated that ginger intake resulted in a significant pain reduction in patients with knee and hip OA [89]. This pain-relieving symptom was also observed in a more recent clinical trial conducted with elderly patients with OA [90]. Similar results were obtained when the ginger extract was administered in nanostructure lipid carriers; as well as relieving pain, the knee joint’s physical function also improved [91]. 

6-gingerol, the main bioactive compound from ginger, was tested in vivo and in vitro to validate its efficacy in IL-1β-induced human OA chondrocytes and in a DMM-induced mouse OA model [92]. The in vivo results revealed that 6-gingerol suppressed cartilage damage, most probably through modulating genes and signaling pathways involved in OA pathogenesis. In vitro studies confirmed this hypothesis as the treatment with 6-gingerol significantly enhanced the Nrf2 expression at protein and mRNA levels. This, in turn, restored the redox status in human OA chondrocytes. In addition, the IL-1β-induced catabolic and inflammatory mediators such as the production of NO, PGE2, and MMP-13 were inhibited [92]. These findings are in conformity with another in vitro trial using ginger extract [108]. 6-gingerol can also stimulate osteoblasts differentiation, as demonstrated in an in vitro study on IL-1β-induced osteoclasts [93].Treatment with 6-gingerol was also found to stimulate osteoblasts’ differentiation via suppressing PGE2 levels and downregulating the expression of RANKL in cytokine, IL-1β-induced osteoclast [93].

### 3.8. Berries (Strawberries and Blueberries)

Strawberries and blueberries contain a wide range of polyphenols such as anthocyanins, phenolic acids, flavonoids, and ellagitannins, which have been implicated for their health-benefiting properties, especially in bone health and in OA via modulating genes, signaling pathways to increase antioxidant status, and ameliorating inflammation [94,95,96,97,98,99,109] (Table 3).

The intake of blueberry was studied in 49 individuals with active OA [94]. Results revealed that 40 g of freeze-dried blueberry powder consumed daily prevented further breakdown and stimulated cartilage repair, accompanied by an improvement in joint flexibility and mobility. This was complemented by an increase in cartilage metabolism serum biomarkers [94]. Similar results were observed in another study where adults between the ages of 45 to 79 with symptomatic knee OA were given 40 g freeze-dried blueberry powder daily for four months. Stiffness and pain decreased significantly, while mobility improved, accompanied by an increased IL-13 concentration and a reduced MCP-1 concentration [95]. In another study, South et al. demonstrated that blueberry powder alleviated pain, reduced mechanical allodynia, and decreased plasma hyaluronic acid levels in an OA monosodium iodoacetate (MIA)-induced rat model [97]. An in vitro study demonstrated the effect of blueberry on proinflammatory cytokines and transcription factor NFκ; blueberry was able to downregulate COX-2, IL-1β, and transcription factor NFκ in rabbits’ synoviocytes stimulated with TNF-α [98].

The consumption of 50g freeze-dried strawberries daily was evaluated for its effect on biomarkers of inflammation and lipid peroxidation in knee OA obese adults. The TNF-α and TNF-R2 were significantly decreased after strawberry consumption [96]. Similar results were obtained in another similar study; strawberry supplementation significantly reduced pain and cartilage degradation, accompanied by a reduction in the serum biomarkers of inflammation (IL-6, IL-1β, and MMP-3) [99].

### 3.9. Herbs and Other Plant Extracts

#### 3.9.1. Silver Fir

Silver fir (*Abies alba* Mill.) is a popular and valuable medicinal plant that has been utilized in the food, pharmaceuticals, and cosmetics industries for its many various health-promoting properties. It is abundant in phenolic acids, lignans, and flavonoids [110,111]. Belinal, a standardized polyphenolic extract of silver fir (*Abies alba* L.) branches, exhibited a significant chondrogenesis effect in bone-derived mesenchymal stem/stromal cells derived from patients with hip OA [106]. In addition, Belinal not only exhibited a higher chondrogenesis effect compared to the untreated but also with resveratrol-treated patients [106]. 

#### 3.9.2. Pine Bark

Maritime pine bark extract or its standardized form Pycnogenol has gained much interest among the scientific community due to its various biological properties [112]. Pycnogenol originates from the bark of the French maritime pine, *Pinus pinaster* and contains between 65% to 75% of procyanidins, coupled with other polyphenols such as phenolic or cinnamic acids and their derivates [112].

Several recent studies have demonstrated the beneficial effects of pine bark extract or Pycnogenol in treating and alleviating symptoms of OA (Table 3). In a review by Rohdewald, the bioactive ingredients in Pycnogenol are said to act synergistically together as many of them exhibit anti-inflammatory actions and free radical scavenging activities. Pycnogenol intake in three different clinical studies resulted in pain alleviation, reduced stiffness, and enhanced mobility [100]. The oral intake of 100 mg of Pycnogenol twice daily for three weeks significantly downregulated the gene expression of MMP3, MMP13, and IL1β in chondrocytes and reduced concentrations of ADAMTS-5 in the serum of the knee OA patients. [107]. In a more extensive clinical study involving 358 OA participants, 120 mg of pine bark extract supplemented with mineral-rich algae significantly alleviated pain and mobility [101]. To ascertain the anti-OA properties of Pine bark polyphenols and rationalize the results of previous clinical studies, a randomized controlled trial was conducted on OA patients who received 200 mg of Pycnogenol per day for three weeks. Analysis of the in vivo distribution of Pycnogenol indicated that some polyphenols and their metabolites were present in the serum and synovial fluid of patients with OA, thereby confirming their functional roles in OA [113].

#### 3.9.3. Boswellia

*Boswellia serrata* extract is a gum resin that has been used in Ayurvedic medicine for centuries to treat a diverse array of health problems. The gum resin, also known as frankincense, contains the chemical markers 1-keto-β-boswellic acid (KBBA) and 3-O-acetyl-11-keto-β-boswellic acid (AKBA). KBBA has been documented to inhibit 5-lipoxygenase, while AKBA suppresses the transcription factor NF-κB [114], thereby indicating its suitability as an anti-inflammatory and antiatherogenic agent in managing the pathogenesis of OA (Table 3).

A standardized oral supplementation of *Boswellia serrata* extract (Boswellin^®^), when given to OA patients for 120 days, significantly improved pain and mobility. In addition, further analysis indicated that the knee joint gap was increased, and osteophytes and serum high sensitive-CRP protein was significantly reduced [102]. A meta-analysis was carried out by Yu et al. on the effects of *Boswellia* in 545 OA patients involving seven studies, and subsequently compared the results with placebo or conventional medicinal treatment. The results revealed that *Boswellia* treatment is an effective and safe treatment option in alleviating pain and improving mobility in OA patients if consumed for at least four weeks [103]. 

AKBA, one of the main ingredients of *Boswellia*, has been demonstrated to play an important role in bone metabolism; AKBA treatment reduced bone loss, enhanced bone formation, and increased osteogenesis. This was accompanied by an increase in mRNA levels of runt-related transcription factor 2 (Runx2), Osterix, OCN, Osteopontin, and ALP. In addition, the GSK-3β/β-catenin signaling pathway was activated [104]. In a recent study, a standardized extract (30%AKBA) of *Boswellia* was demonstrated to reduce oxidative stress (via reducing NO synthase) and decrease levels of proinflammatory mediators such as TNF-α, IL-6, and COX-2. Furthermore, the matrix proteins were also shown to be preserved via the enhanced hyaluronan levels in synovial fluid. The levels of phosphorylated-NF-κB (P65) were also reduced, coupled with the inhibition of enzymes such as collagenase, elastase, and hyaluronidase enzymes [105].

## 4. Conclusive Remarks and Future Perspectives

The studies discussed in this review have assessed individual polyphenols and extracts rich in polyphenols in vitro and in vivo in various OA models. Most clinical, pre-clinical, and clinical studies reveal that polyphenols exert pain-relieving symptoms coupled with increased functional capacity in OA models. The main aim was to investigate the mechanisms underlying their beneficial effects on pathophysiological processes of OA etiology, especially those carried out in the last five years. These investigations have elucidated that polyphenols can modulate OA pathogenesis through the following mechanisms:

(a) Inhibiting apoptosis—via
  - Downregulating/inhibiting signaling pathways such as the IRAK1/NLRP3; TLR4/NF-κB; Wnt/β-Catenin.
  - Suppressing the expression of matrix-degrading proteases (Caspase-3, Caspase-9, MMP-1, MMP-3, and MMP-13), protein kinase (mTOR, P_38_), and aggrecanase (*ADAMTS4 and ADAMTS 5).*
  - Suppressing the expression inflammatory mediators ((IL-1β, IL-6, IL-16, IL-6 IL-17, and TNF-α).
  - Reducing ROS—suppression of NO synthase, 5-LOX, COX-1, and COX-2; increasing levels of antioxidant enzymes such as SOD, CAT, and GPX.
  - Upregulating SOX9 and downregulating RANKL expression.
(b) Repair articular cartilage damage via
  - Increasing chondrogenesis—through upregulating expression of miR140-3p.
  - Increasing the glycosaminoglycan synthesis, collagen (upregulation of COL-II), and aggrecan (upregulation of ACAN).
  - Protect against oxidative stress through increasing M2 macrophage, HO-1 production, and activating Nrf-2.
  - Increasing autophagy through (a) suppressing miRNA-34a that upregulates signaling pathways—E2F1/PITX1 and inhibition of Akt/mTOR, (b) enhancing the expression of SIRT 1, (c) increasing the levels of Beclin1, LC3, and Coll II, (d) maintaining the chondrocyte phenotype by downregulating the GSK-3β/β-catenin signaling pathway.

These diverse underlying mechanisms of action indicate that polyphenols can potentially play a vital role in the future development of effective and safe therapeutic agents in managing OA. However, due to the complexity of OA pathogenesis, the efficacy of using a single polyphenol to treat OA may be limited. The future polyphenol-based approaches may require a combination of compounds that have complementary or synergistic effects and can achieve multiple molecular targets to exert a more profound impact on OA. In addition, efforts should be aimed at utilizing nanotechnology to design and develop new delivery systems to improve bioavailability and attain site-specific targeting for enhanced efficacy.

## Figures and Tables

**Table 1:** Efficacy and mechanism of action of quercetin, resveratrol, and curcumin in managing osteoarthritis.

Polyphenol	Clinical Effects	Pre-Clinical Effects	In Vitro Effects	Mechanism of Action
Quercetin	Not reported	↓ cartilage degradation, inflammation and oxidative stress in rat OA model [46] ↓ cartilage degradation, apoptosis of chondrocytes in rat OA model [47] ↓ degradation of the cartilage in MIA-induced knee mouse OA model [49] ↑ SOD and TIMP-1, ↓MMP-13, ↓ degeneration of OA through weakening the oxidative stress responses in a rabbit knee OA model [50]	↓ inflammation and apoptosis in IL-1β-induced rat chondrocytes [46,48] ↓ matrix-degrading proteases and reverse cartilage matrix degradation in IL-1β-induced rat chondrocytes [47]	↓ IRAK1/NLRP3; ↓caspase-3 expression; ↓ proinflammatory cytokines (IL-1β, IL-18, and TNF-α) [46] ↓ Caspase-3 pathway; ↑ TGF-β1 and TGF-β2 in the synovial fluid; ↑the ratio of M2 macrophages in the synovial membrane, ↑ synthesis of glycosaminoglycan [47] Inhibit the P38 MAPK signaling pathway [48] ↓MMP-3 and MMP-13 in the blood [49]
Resveratrol	Reduced OA-associated pain [5,51]	↓ inflammatory damage and protected against OA in rat OA model [52]	↓ IL-1β-induced inflammation in SW1353 chondrocytes cells [52] ↓inflammation in Osteoarthritis (OA)-Like Chondrocyte Cell Model [53] ↓ oxidative stress-induced response in human chondrocytes [35] ↓ IL-1 and IL-1β mRNA expression and ↓ production of ROS and NO induced by monosodium urate and calcium pyrophosphate crystal [54] ↓ production of ROS and NO [54]	Inhibited TLR4/NF-κB signaling Pathway [52] ↓ expression of TNF-α, IL-1β, IL-6, and IL-18; ↓caspase-3/9 activity; ↓ Inducible NO synthase; ↓ expression of nuclear factor (NF)-κB; ↑ expression of HO-1 and Nrf-2 [55] ↓ serum levels of IL-1 β, IL-6, IL-17, TNF-α, and MCP-1; ↓ protein levels of TLR-4, and NF-κB p65; ↓ caspase-9 Bax protein levels [56] H_2_O_2_-induced free radicals, inhibition of TNF-α, ↓ IL-1β, and MMP-1 [53] ↑ expression SIRT1 and ↑ Erk1/2 phosphorylation in subchondral bone, ↑ mineralization of OA osteoblasts [57] ↓expression of leptin [57]
Curcumin/curcuminoid	Alleviated pain and discomfort of OA [58,59] and increased functional capacity with better tolerability [60]	Ameliorated the pathophysiology ↓ joint degradation and exhibited chondroprotective effect in OA-induced rat model [61,62,63,64] Intra-articular injections of curcumin precursor ↓ cartilage damage and regulated subchondral bone alteration in an OA knee rat model [65]	↓ oxidative stress-induced-endoplasmic reticulum (ER) stress and degradation of TBHP-stimulated chondrocytes cells [63] ↓ IL-1β-induced inflammation [64]	Inhibited apoptosis via ↑ E2F1/PITX1; ↑ autophagy via the Akt/mTOR pathway by ↓ miRNA-34a [61] ↓ levels of TNF-α, IL-1β, IL-6, COMP, and CRP; ↓ expressions of MMP-3, 5-LOX, COX-2, and NFκB in synovial tissue of rats; ↓ serum MDA level; ↑ levels of antioxidant enzymes SOD, CAT, and GPX [62] ↑ expression SIRT1 [63] ↓ expression of COLIIa and SOX9; ↓ overexpression of iNOS and COX-2 and suppress the activation of the NF-κB/HIF-2α pathway [64] ↓ levels of TNF-α and ↓synovial inflammation [65]

CAT, catalase; COL- II, collagen II; COX, cyclooxygenase, CRP, C-reactive protein; E2F1, eukaryotic translation termination factor 1; GPX, glutathione peroxidase; HO-1, heme oxygenase-1; iNOS, inducible nitric oxide synthase; IRAK1, Interleukin-1 receptor-associated kinase 1; IL, interleukin; 5-LOX, 5-lipoxygenase; mTOR, mammalian target of rapamycin; MAPK, mitogen-activated protein kinase; MMPs, matrix metalloproteinases, MIA, monosodium iodoacetate; NF-κB, nuclear factor-*κB*; *NLRP3*, nucleotide-binding domain, leucine-rich repeat, pyrin domain-containing 3; NO, nitric oxide; Nrf-2, nuclear factor erythroid 2-related factor 2; OA, osteoarthritis; PITX1, paired-like homeodomain transcription factor 1; SIRT 1, sirtuin 1; SOD, superoxide dismutase; SOX9, SRY-Box Transcription Factor 9; p38 mitogen-activated protein kinases; TGF-β, transforming growth factor; TNF-α, tumor necrosis factor alpha; TLR4, Toll-like receptor 4; ↑, increase; ↓, decrease.

**Table 2:** Efficacy and mechanism of action of EGCG and green tea polyphenols, rosmarinic acid, and genistein in managing OA.

Polyphenol	Clinical Effects	Pre-Clinical Effects	In Vitro Effects	Mechanism of Action
EGCG and green tea polyphenols	Reduced pain and improved functional capacity of the knee joint [73]	Intra-articular injection improved functional capacity, ↓ inflammation and cartilage degradation in posttraumatic mice OA model [70] ↓ cartilage degradation, proteoglycan loss and articular surface erosion in an aging-related OA model in guinea pigs [71] ↓ knee joint damage in rabbit OA model [74] ↓ OA progression, ↓ cartilage loss in surgically-induced mouse OA model [75] ↓ symptoms caused by gout [77]	↓ chondrocyte senescence and ↓ the inflammatory response in IL-1β-stimulated human chondrocytes [71] ↑ cartilage regeneration in IL-1β-induced OA [75] ↓ inflammation caused by calcium pyrophosphate crystals [76]	↓ COX-2 and MMP-13 in synovial tissue and cartilage; ↓ expression of the mTOR; ↑ LC 3 and, Beclin1 and p62 causing upregulation of autophagy [70] ↑ COL-II; ↓ COX-2, IL-1β 1, MMP-13, p16^Ink4a^ [71] ↑ expression of human miR140-3p and ↓ ADAMTS5 expression in human OA chondrocytes [72] ↓ NO production [74] ↓ expression of IL-1β, COX-2, TNF-α, MMP1, MMP13, ADMTS5; ↑ gene expression of COL-II, SOX9, ACAN [75]
rosmarinic acid	Not reported	↓ swelling and joint diameter in adjuvant-induced arthritic rats [78]	↓ oxidative stress-induced responses in IL-1β-induced chondrocytes [79] ↑ differentiation of chondrocytes in rabbit articular chondrocytes [80] ↓ chondrocyte senescence [81]	↓ levels of TNF-α; ↑ GSH and SOD [78] ↓ the MMP-1, MMP-3, and MMP-13 expression; ↓ NO and PGE_2_ production; inhibit p38 and p65 translocation in rat articular chondrocytes [79] ↑ COL-II, sulfated-proteoglycan, COX-2, and PGE_2_ production in rabbit articular chondrocytes, ↑ the ERK-1/2, and p38 kinase signaling pathways [80] ↓ production of IL-6; ↓gene and protein expression of ADAMTS-4 and ADAMTS-5; stopped inhibition of ACAN and COL2 gene expression in IL-1β-induced chondrocytes [81]
genistein	Not reported	↓ progression of OA, ↓ chondrocyte apoptosis slowing cartilage degeneration in OA-induced rat model [82,83,84] reversed the condyle cartilage damage in OA-induced rat model [84]	↓ inflammation and oxidative stress in IL-1β-stimulated human OA chondrocytes [82]	Inhibited NO synthase-2; ↓COX-2 expression in OA chondrocytes, ↓ MMP-1, MMP-2, MMP-3, and MMP-13, stimulated Ho-1 expression, and activation of the Nrf-2 pathway; ↓ level of IL-1β, IL-6, and TNF-α in the serum [82] ↓ TNF-α and IL-1β in the synovial fluid; ↑ collagen and acid glycosaminoglycan content; ↓ the expression of caspase 3 [83] ↓ expression of p65 and inflammatory cytokines (IL-1β and TNF-α) [84]

ACAN, aggrecan; ADAMTS5, ADAM Metallopeptidase with Thrombospondin Type 1 Motif 5; COL-II, collagen type II; COX, cyclooxygenase; HO-1, heme oxygenase-1; IL, interleukin; mTOR, mammalian target of rapamycin; LC3, microtubule-associated protein *1* light chain 3, MMPs, matrix metalloproteinases, NF-κB, nuclear factor-κB; NO, nitric oxide; Nrf-2, nuclear factor erythroid 2-related factor 2; OA, osteoarthritis; SOD, superoxide dismutase; SOX9, SRY-Box Transcription Factor 9; p38 mitogen-activated protein kinases; TNF-α, tumor necrosis factor-alpha; ↑, increase; ↓, decrease.

**Table 3:** Efficacy and mechanism of action of ginger and gingerol, berries, Silver fir, Pine bark, and *Boswellia* in managing OA.

Type of Plant Extracts	Clinical Effects	Pre-Clinical Effects	In Vitro Effects	Mechanism of Action
Ginger and gingerol	Reduced pain in patients with OA [89,90,91] and improved the knee joint physical function [91]	Preserve cartilage, ↓ cartilage damage in mouse OA-induced by DMM [92]	↓ inflammation and oxidative stress in IL-1β-stimulated human OA chondrocytes [92]	↑ Nrf2 expression at protein (GSTA4-4) and mRNA levels; inhibit NO, PGE2, and MMP-13 [92] ↓ PGE2 synthesis and ↓ the expression of RANKL to stimulate osteoblasts differentiation [93]
Berries (blueberry and strawberry)	Blueberry inhibited degradation of cartilage and simulated repair [94]; alleviated pain, reduced stiffness, improved joint flexibility and mobility [94,95] Strawberry reduced inflammation and lipid peroxidation [96], alleviated pain and cartilage degradation [96]	Blueberry ↓ pain, ↓ mechanical allodynia in an OA monosodium iodoacetate-induced rat model [97]	Blueberry ↓ inflammation in rabbit synoviocytes-induced TNF-α [98]	Blueberry ↑ cartilage metabolism serum biomarkers [94] ↑ IL-13 concentration ↓ MCP-1 concentration [95] ↓ plasma hyaluronic acid levels [97] ↓ proinflammatory cytokines and transcription factor NF-κB [98] Strawberry ↓ TNF-α, TNF-R2 [96] ↓ IL-6, IL-1β, and MMP-3 [99]
Other plant extracts (Silver fir, Pine bark, and *Boswellia*)	Pycnogenol/pine bark extract reduced pain, stiffness, and enhanced mobility in OA patients [100,101] *Boswellia* alleviated pain and mobility in OA patients [102,103]	*Boswellia* treatment ↓bone loss, enhanced bone formation in titanium-induced mouse calvaria model [104] *Boswellia* treatment improved bone architecture and articular cartilage with open joint space in collagen-induced arthritis in rats [105].	Silver fir extract (Belinal) exhibited chondrogenesis effect in bone-derived mesenchymal stem/stromal cells from patients with hip OA [106]	Pycnogenol/ pine bark extract ↓ MMP3, MMP13, and IL1β gene expressions and ↓ serum ADAMTS-5 [107] *Boswellia* ↓serum CRP protein [102] ↑ mRNA levels of Runx2; activated the GSK-3β/β-catenin signaling pathway [104] ↓ NO synthase; ↓TNF-α, IL-6, and COX-2; ↑ hyaluronan levels in synovial fluid; ↓ phosphorylated-NF-κB (P65); inhibition of collagenase, elastase, hyaluronidase enzymes [105]

ADAMTS5, ADAM Metallopeptidase with Thrombospondin Type 1 Motif 5; COX, cyclooxygenase, CRP, C-reactive protein; DMM, destabilization of medial meniscus; G*SK*-*3β*, glycogen synthase kinase-3β (); IGF-1, insulin-like growth factor *1*; IL, *Interleukin*; MCP, membrane cofactor protein; MMPs, Matrix metalloproteinases; NF-κB, nuclear factor-*κB*; NO, nitric oxide; Nrf-2, nuclear factor erythroid 2-related factor 2; OA, osteoarthritis; PGE2, Prostaglandin E2; RANKL, receptor activator of nuclear factor kappa B ligand; Runx2, runt-related transcription factor *2*; TNF, tumor necrosis factor; ↑, increase; ↓, decrease.

## Data Availability

Not applicable.
